# Assessment of suicidal risk using Minnesota multiphasic personality inventory-2 restructured form

**DOI:** 10.1186/s12888-020-02495-2

**Published:** 2020-02-26

**Authors:** Sunhae Kim, Hye-Kyung Lee, Kounseok Lee

**Affiliations:** 1 Department of Psychiatry, Hanyang University Medical Center, 222-1, Wangsimni-ro, Seongdong-gu, Seoul, 04763 Republic of Korea; 2 Department of Nursing, College of Nursing and Health, Kongju National University, 56 Gongjudaehak-ro, Gongju, 32588 Republic of Korea

**Keywords:** Minnesota multiphasic personality inventor-2 restructured form, Suicide, Risk factors

## Abstract

**Background:**

Suicide is a major social issue, affected by both social and psychopathological factors. This study investigated suicide risk assessment using the Minnesota Multiphasic Personality Inventory-2 Restructured Form (MMPI-2-RF).

**Methods:**

Data were collected from 7824 college students using the MMPI-2-RF. The participants were classified into high-, moderate-, and low-risk for suicide groups based on their scores on the structured Mini-International Neuropsychiatric Interview (MINI) for comparative analysis. The relationships between scores on the Restructured Clinical (RC) Scales of the MMPI-2-RF and suicide risk level were investigated using a multiple logistic regression.

**Results:**

Out of the 7824 participants, 964 (12.3%) were identified as being at risk of suicide. There were 553 participants considered low-risk, 312 moderate-risk, and 99 at high-risk. Suicide risk in the participants tended to increase as RC scale scores increased. Out of the nine RC scales, the Demoralization (RCd) and Negative Emotions (RC7) scale scores were highest across all risk groups. The results of a multiple logistic regression indicated that the Demoralization (RCd) scores were significantly elevated in all three suicide risk groups. Antisocial Behavior (RC4) and Aberrant Experiences (RC8) scale scores were significantly elevated for the low-risk group, whereas Somatic Complaints (RC1) scores were elevated for the moderate-risk group, and Somatic Complaints (RC1), Low Positive Emotions (RC2), Antisocial Behavior (RC4), and Ideas of Persecution (RC6) scale scores were elevated for the high-risk group.

**Conclusions:**

Compared to the healthy control group, all three suicide risk groups showed elevated scores on the RC Scales overall, suggesting that various psychopathological factors are involved in the etiology of suicide. More psychopathologic factors were found to influence suicide-related issues in the higher risk groups than lower risk groups, suggesting that more risk factors are involved in higher suicide risk groups. Compared to healthy controls, even the low-risk group showed a significant elevation in emotional factors and antisocial behaviors. While the healthy controls and those at risk of suicide differed significantly on both the Demoralization (RCd) and Negative Emotions (RC7) scales, only the Demoralization (RCd) scale appeared to be able to screen for high suicide risk.

## Background

According to health statistics released by the Organization for Economic Co-operation and Development (OECD) in 2018, Korea ranked second with 53.0 suicides per 100,000 people in 2017, and according to the OECD age-adjusted suicide rate, Korea again ranked second with 23.0 suicides (per 100,000 of the OECD standard population) [1]. Because of national policies and interventions to reduce the rate of suicide, which is a serious cause of death in Korea, the number of suicides decreased by 4.8% in 2017 when compared with the number in 2016. However, the suicide rate for those under the age of 15 years increased by 66.7% in the same year, and suicide has remained the top cause of death among teenagers and those in their 20s and 30s. Unfortunately, the suicide mortality rate in Korea, which had been declining steadily over the past few years, increased by 2.3 suicides (9.5%) in 2018 when compared with the value in 2017. Compared with the OECD average age-adjusted suicide rate of 11.5, Korea recorded a higher rate of 24.7, ranking at the top among OECD countries. In particular, the suicide rate increased in individuals of all ages, except those aged 80 years or older, and it was high in teenagers (22.1%) and those in their 40s (13.1%) and 30s (12.2%) [1]. Considering that a history of suicidal ideation and/or suicidal attempt is the biggest risk factor for suicide, youth with a history of suicidal behavior present a high-risk group with potential for suicide [2]. Importantly, a survey conducted by the American College Health Association in 2015 found that 1.5% of students reported at least one suicide attempt, and 9.8% of the students considered suicide seriously at least once during the past year. In Korea, 4.1% of those aged 19 or older reported having seriously considered dying by suicide [2]. Over the past 50 years, suicide has increased in those aged between 15 and 24 years old, with over 1000 suicides annually. Suicide was the second most common cause of death for the population during this time. Therefore, identifying suicide risk factors for early adults is of particular importance [1].

Suicide risk assessment is challenging; it is particularly difficult to accurately predict the probability of suicide attempt and death by suicide [3]. Understanding suicide risk requires comprehensive evaluation of an individual’s intrapersonal and interpersonal characteristics. The key intrapersonal characteristics include clinical diagnoses, such as depression, anxiety, personality disorders, and substance abuse [4]. Suicide is influenced by a wide range of psychopathologies, and in over 90% of those who commit suicide, a complex interaction of psychiatric disorders, including mood disorders, substance abuse disorders, anxiety disorders, and personality disorders, precedes suicide [5, 6]. Therefore, it is crucial to grasp the characteristics of various types of psychopathologies. Minnesota Multiphasic Personality Inventory-2 (MMPI-2) is widely used in suicide risk research to assess the emotional state and personality of patients in clinical settings [7–9]. However, studies conducted with the MMPI-2 Clinical Scales have yielded inconsistent findings, including the relationships between suicidal ideation and behavior and elevations of Scales 3 (Hysteria), 4 (Psychopathic Deviate), 7 (Psychasthenia), 9 (Hypomania), and 0 (Social Introversion) in addition to Scales 2 (Depression), 6 (Paranoia), and 8 (Schizophrenia) [9–13]. Also in Korean studies analyzing MMPI-2 results of patients with major depression, only the 6 (Paranoia) clinical scale was shown as ‘high’ in those with a suicidal attempt history as compared to those without such history [14]. Seo et al. reported that the MMPI-2 clinical scales 6 (Paranoia), 8 (Schizophrenia), and 9 (Hypomania) were noted as significantly high in 75 patients who attempted suicide as compared to 115 people in the control group [15].

The poor discriminatory power of the MMPI-2 scales in suicide risk assessment is due to the high correlations among the MMPI-2 Clinical Scales [16]. Notably, the demoralization factor is scattered around Clinical Scales and elevates multiple scales together, making it difficult to interpret profiles accurately. Therefore, the demoralization factor was removed from the scales to measure the original key elements, and the “restructured” Clinical Scales became the Restructured Clinical (RC) Scales of the MMPI-2-RF [16, 17]. The Restructured Clinical Scales shows improved convergent validity and discriminant validity to the existing clinical scale. We confirmed that the revised clinical scale aids greatly in resolution of analytical ambiguity questions that are raised regarding the clinical scales. And the RC scales of the MMPI-2-RF can likely indicate an individual’s overall intrapersonal characteristics and increase the likelihood of diagnosing pathologies [16, 18, 19]. Which are required to evaluate the suicide risk because they describe the individual’s overall functional level according to the center points of all measurements, in addition to the three higher-order (H-O) scales of the measure [20].

Among MMPI-2-RF scales, RC scales have been found to have higher internal consistency and lower interrelationship when compared with clinical scales, and evidence regarding their validity is accumulating from empirical studies conducted in various settings, such as mental health outpatient clinics and wards, individual counseling settings, university counseling centers, drug addiction treatment centers, and correctional facilities [16, 21–27]. Some previous studies suggested that MMPI-2-RF could be used to assess suicide risk [28, 29]. The previous study on psychiatric outpatients reported that the interaction of RCd and RC9 differed between patients with suicide attempts and patients with no attempts, but both groups had suicidal ideation. It also reported that the high score of RCd and RC9 had a static correlation with suicide attempt [30].. Another on the usefulness of RF scales demonstrated that the Emotional/Intenalizing Dysfunction (EID), Behavioral/Externalizing Dysfunction (BXD), Low Positive Emotions (RC2), hypomanic activation (RC9), Helplessness/Hopelessness (HLP), Anxiety (AXY), and Suicidal/Death Ideation (SUI) scales had significant explanatory power for suicide risk [8]. In addition, the SUI, RCd and RC2 scales showed a significant correlation with the history of suicidal attempts, the history of suicidal ideation, recent suicidal ideation, and suicide risk information from patient interviews which included details for suicide attempts in the last month [31].

### Objectives

In this research, we investigated the possibility of screening for suicide risk using the MMPI while reflecting the limitations of repeated investigations in the literature on suicidal risk and the MMPI-2-RF, suicidal tendency and classification of risk groups using suicidality module of MINI.

Suicidal ideation is used to predict suicide risk because it is considered to be on spectrum continuum associated with suicide attempt and suicide, and in particular, individuals with high suicidal ideation might have multiple suicide attempts [32, 33].

We identified the pathological personality types that have the greatest effect on suicidal risk groups among the MMPI-2-RF RC total scale to compensate for the drawbacks described in prior research which showed the relationship between suicidal risk utilizing only the RCd and RC9 scales (emotional pain indicators based on depression/hopelessness), and furthermore to identify cutoff values for the psychological levels of intrapersonal characteristics using Receiver Operating Characteristic (ROC) curve analysis.

## Methods

### Study participants

This study used the data from the Capacity Building Project conducted in Kongju National University. All participants were students, the confidentiality of the results explained and the use of their survey responses for research, and they provided written consent. The study analyzed the answers given by 7824 students out of a total of 8769, excluding 945 students (3685 men and 4139 women) were included in the data analyses. The study was approved by the institutional review board of Kongju National University.

### Measures

#### Minnesota multiphasic personality inventory-2-restructured form (MMPI-2-RF)

To confirm the psychological characteristics associated with suicide risk, we used the Minnesota Multiphasic Personality Inventory-2-Restructured Form (MMPI-2-RF) released in 2011. The measure involves a questionnaire with a total of 338 items, each of which is answered “Yes” or “No,” and there are a total of 50 scales to effectively measure the clinical meaning of MMPI-2 items. The 50 scales of MMPI-2-RF have a hierarchical structure, and they were developed to minimize conceptual redundancy among the scales. They include eight validity scales, 42 main scales (three H-O scales, nine RC scales, 23 specific problem scales, two interest scales, and PSY-5 [personality psychopathology five] scales). This study focused on the nine RC scales [RCd (Demoralization), RC1(Somatic Complaints), RC2(Low Positive Emotions), RC3(Cynicism), RC4(Antisocial Behavior), RC6(Ideas of Persecution), RC7(Dysfunctional Negative Emotions), RC8(Aberrant Experiences), RC9(Hypomanic Activation)] for analysis. We used the Korean version of the MMPI-2-RF [18].

#### Suicide risk assessment

Suicide risk was assessed using the suicide risk assessment module of the Korean version of the Mini International Neuropsychiatric Interview (MINI). The MINI is a structured interview tool developed in 1998 for the diagnosis of Axis I disorders of DSM-IV and ICD-10. It has verified a minimum 0.70 specificity and a 0.85 sensitivity in MINI in clinical interview situations such as the Structured Clinical Interview for the DSM, throughout named psychological disorders [34].

In this study, the Korean version 5.0.0 was used, which was standardized [35]. Among these, suicidal tendency was assessed using the suicidal tendency module developed by Sheehan et al., which includes the following six questions related to “wish for death” with varying weights [36]. We followed 307 patients discharged from the psychiatric ward for 1 year. We analyzed the results using the MINI scale to find a potential predictor of suicide attempt. The subjects were categorized in four groups (no symptoms, suicidal behavior, suicidal behavior and NSSI, and NSSI only) including a group with suicidal behavior and a non-suicidal self-harm group (NSSI). The total score of the MINI showed a significant correlation to the self-harm group when stratified by age, sex and psychiatric diagnosis. We divided the patients into a moderate-risk group and a high-risk group using the MINI score 6 (or more than two positive items) and score 10 (or more than three positive items) as criteria. These criteria showed good sensitivity (0.61–0.75) and specificity (0.61–0.75) established from the patient history of self-harm acts. When each group was considered, the MINI suicidal sub-scale score was a good predictor in two groups with suicidal behavior, but not in the NSSI group [37].

Current suicidal risk is assessed using six items. A response of “Yes” to at least one of the six items is considered as being at risk of suicide. Based on the sum of the weighted scores of the “Yes” items, 1–5 points are classified as low-risk, 6–9 points as moderate-risk, and 10 points and higher as high-risk. In this study, the low-, moderate-, and high-risk groups were compared with one another and with the controls, whose total suicide risk scores were 0.

#### The Korean version of the patient health questionnaire-9 (PHQ-9)

This depression screening tool is a self-report questionnaire [38, 39]. The instrument includes nine items that correspond to the nine DSM-IV diagnostic criteria for major depressive episode. Each item is scored 0–3, and the total score ranges between 0 and 27. In this study, the Korean version of the PHQ-9 with established reliability and validity since its adaptation into Korean in 2010 was used [40]. Cronbach’s alpha in this study was 0.822.

### Statistical analysis

Demographic variables were analyzed using t-tests and χ^2^ tests. The differences between the three suicide risk groups on the MMPI-2-RF RC Scales were tested using analysis of variance (ANOVA). To lower the probability of type 1 error, we tested significance with a *p*-value <.01 and used Scheffé’s method as a post-hoc test. The relationships between the RC Scales and the different suicide risk groups were tested using multiple logistic regression. We performed a multinomial logistic regression analysis instead of individual logistic regression analyses, and we categorized and compared the suicide risk groups according to low, medium, and high risk. Furthermore, the reference group of the multinomial logistic regression analysis was set as the control group and was compared with the other groups according to the suicide risk level. To determine how well the Demoralization (RCd) and Negative Emotions (RC7) scales, which showed the largest differences for those at risk of suicide, discriminated between the suicide risk groups, receiver operating characteristics (ROC) curve analysis (MedClac version 18.10.2) was performed and the sensitivity and specificity of individual cutoff scores were examined. Statistical analysis was performed using the SPSS 24.0 (IBM corp., Armonk, NY, USA).

## Results

### Demographic characteristics

Out of the 7824 participants (age range: 17~48), 964 (12.3%) were endorsed at least one of the suicide risk items on the MINI. The mean age of the suicide risk group was 19.61 years (SD = 1.1) and the group included 613 (63.6%) female students. While the group’s mean age was not significantly different from that of controls, the female ratio was higher than in the control group (51.4%, *N* = 3526). The 964 subjects at risk of suicide were classified into the low-risk group (57.4%), moderate-risk group (32.4%), and high-risk group (10.3%) (Table 1).

**Table 1:** General characteristics of the participants (*n* = 7824)

	Suicidal risk group (*n* = 964)	Control (*n* = 6860)	t or *x*^2^	*P*-value
Age	19.61 ± 1.1	19.57 ± 1.3	0.854	0.393
Sex			50.407	< 0.001
Male	351(36.4%)	3334(48.6%)		
Female	613(63.6%)	3526(51.4%)		
PHQ-9 score	8.46 ± 5.2	3.9 ± 3.4	36.517	< 0.001
Suicide risk				
Low	553(57.4%)			
Medium	312(32.4%)			
High	99(10.3%)			

Values were presented as mean ± SD or n (%)

### Analysis of MMPI-2-RF RC scales by suicide risk group

The MMPI-2-RF scores of the low-risk group, the moderate-risk group, and the high-risk group were analyzed to determine potential differences between the groups.

The three groups’ scores were significantly elevated on all nine RC Scales (*p* < 0.001), but most elevated on the Demoralization (RCd) and Emotions (RC7) scales. The RC Scale scores were higher in the groups with higher suicide risk.

**Fig. 1:**
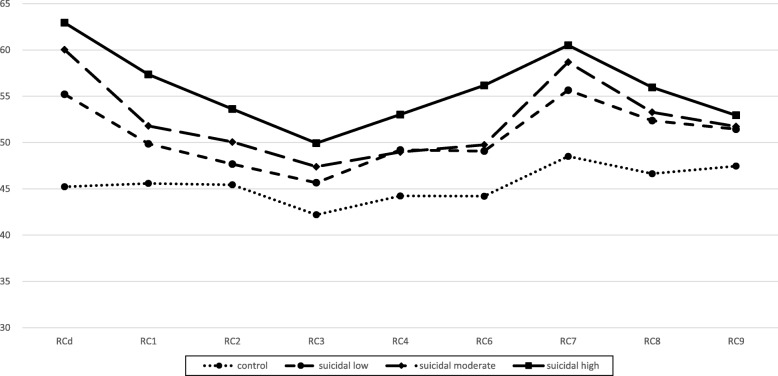
The profiles of the MMPI-2-RF restructured clinical (RC) scales among the 3 suicidal risk groups and control group

The Demoralization (RCd), Somatic Complaints (RC1), Low Positive Emotions (RC2), Cynicism (RC3), and Negative Emotions (RC7) scale scores were significantly higher in the moderate-risk group than the low-risk group; all the RC Scale scores except Hypomanic Activation (RC9) were significantly higher in the high-risk group than the low-risk group (*p* < 0.001); the Somatic Complaints (RC1), Low Positive Emotions (RC2), Cynicism (RC3), Antisocial Behavior (RC4), and Ideas of Persecution (RC6) scale scores were significantly higher in the high-risk group than in the moderate-risk group (Table 2, Fig. 1).

**Table 2:** Summary of descriptive statistics for MMPI-2-RF restructured clinical (RC) scales among suicidal risk groups

	Control (n = 6860)	Suicidal Low risk (*n* = 553)	Suicidal Moderate risk (*n* = 312)	Suicidal High risk (*n* = 99)	F	*P*	d1	d2	d3	d4	d5	d6
RCd	45.22 ± 10.200	55.22 ± 12.763	60.04 ± 13.479	62.95 ± 13.875	405.747	< 0.001	10.002^b^	14.816^b^	17.727^b^	4.814^b^	7.725^b^	2.911
RC1	45.59 ± 7.582	49.87 ± 9.309	51.80 ± 10.469	57.36 ± 13.144	169.700	< 0.001	4.282^b^	6.219^b^	11.778^b^	1.936^a^	7.496^b^	5.559^b^
RC2	45.44 ± 8.140	47.67 ± 9.613	50.07 ± 11.058	53.63 ± 12.744	67.657	< 0.001	2.230^b^	4.630^b^	8.189^b^	2.400^b^	5.959^b^	3.559^a^
RC3	42.20 ± 7.255	45.66 ± 8.338	47.39 ± 9.396	49.94 ± 11.829	110.107	< 0.001	3.464^b^	5.192^b^	7.740^b^	1.727^a^	4.276^b^	2.548^a^
RC4	44.24 ± 8.000	49.21 ± 9.370	48.98 ± 8.288	53.03 ± 10.223	125.715	< 0.001	4.967^b^	4.742^b^	8.794^b^	0.234	3.189^b^	4.053^b^
RC6	44.20 ± 8.185	49.08 ± 9.938	49.76 ± 9.991	56.17 ± 12.547	152.973	< 0.001	4.872^b^	5.552^b^	11.967^b^	0.680	7.096^b^	6.415^b^
RC7	48.51 ± 9.409	55.66 ± 11.155	58.69 ± 11.295	60.52 ± 12.584	235.133	< 0.001	7.143^b^	10.176^b^	12.002^b^	3.033^b^	4.859^b^	1.826
RC8	46.64 ± 8.224	52.37 ± 9.930	53.28 ± 11.133	55.96 ± 12.783	162.141	< 0.001	5.726^b^	6.641^b^	9.319^b^	0.915	3.593^b^	2.678
RC9	47.46 ± 8.417	51.44 ± 9.457	51.74 ± 9.003	52.95 ± 11.605	69.810	< 0.001	3.985^b^	4.286^b^	5.491^b^	0.301	1.506	1.206

^a^*P* < 0.01

^b^*P* < 0.05

Scheffé ANOVA post hoc test was used. *MMPI-2-RF* Minnesota multiphasic personality inventory–2–restructured form, *RCd* Demoralization, *RC1* Somatic complaints, *RC2* Low positive emotions, *RC3* Cynicism, *RC4* Antisocial behavior, *RC6* Ideas of persecution, *RC7* Dysfunctional negative emotions, *RC8* Aberrant experiences, *RC9* Hypomanic activation, *d1* Control vs suicidal low risk group, *d2* Control versus suicidal moderate risk group, *d3* Control versus suicidal high risk group, *d4* Suicidal low risk group versus suicidal moderate risk group, *d5* Suicidal low risk group versus suicidal high risk group, *d6* Suicidal moderate risk group versus suicidal high risk group

### Regression analysis for suicide risk and MMPI-2-RF scale scores

Table 3 shows the results of effects of the suicide risk groups on the RC Scale scores adjusted based on the control group. Odds ratios (ORs) for the Demoralization (RCd) across all three groups were significant: 1.067 (95% CI 1.054–1.080) for the low-risk group, 1.088 (95% CI 1.072–1.105) for the moderate-risk group. The odds ratio was 1.085 (95% CI 1.057–1.114) for the high suicide risk group, which indicates greater risk when compared with that for the control group. At the *P* value of 0.001, the most significant ORs were 1.061 (RC1, 95% CI 10.37–1.085) in the high-risk group and 1.030 (RC4, 95% CI 1.017–1.042) in the low-risk group.

**Table 3:** The association between MMPI-2-RF restructured clinical (RC) scales and suicidal risk group (the result of multiple logistic regression analysis)

	Suicidal Low risk group		Suicidal Moderate risk group		Suicidal High risk group	
	OR (96% CI)	*p* value	OR (96% CI)	*p* value	OR (96% CI)	*p* value
RCd	1.067(1.054–1.080)	< 0.001*	1.088(1.072–1.105)	< 0.001*	1.085(1.057–1.114)	< 0.001*
RC1	1.008(0.995–1.020)	0.224	1.021(1.005–1.036)	0.007*	1.061(1.037–1.085)	< 0.001*
RC2	0.996(0.983–1.009)	0.573	1.007(0.991–1.024)	0.377	1.030(1.005–1.056)	0.017*
RC3	0.990(0.976–1.004)	0.165	1.007(0.990–1.024)	0.422	1.006(0.979–1.034)	0.654
RC4	1.030(1.017–1.042)	< 0.001*	1.016(1.000–1.032)	0.057	1.029(1.004–1.055)	0.023*
RC6	0.996(0.983–1.009)	0.537	0.984(0.968–1.001)	0.065	1.031(1.005–1.056)	0.017*
RC7	0.994(0.979–1.009)	0.430	1.000(0.981–1.019)	0.983	0.977(0.946–1.009)	0.152
RC8	1.015(1.002–1.029)	0.028*	1.008(0.990–1.025)	0.391	0.984(0.957–1.012)	0.250
RC9	1.006(0.991–1.020)	0.459	1.007(0.987–1.026)	0.505	1.015(0.983–1.049)	0.350

*OR* Odds ratio, *CI* Confidence interval, *RCd* Demoralization, *RC1* Somatic complaints, *RC2* Low positive emotions, *RC3* Cynicism, *RC4* Antisocial behavior, *RC6* Ideas of persecution, *RC7* Dysfunctional negative emotions, *RC8* Aberrant experiences, *RC9* Hypomanic activation

*: statistically significant

### Diagnostic power of demoralization (RCd) and negative emotions (RC7) for suicide risk groups

**Fig. 2:**
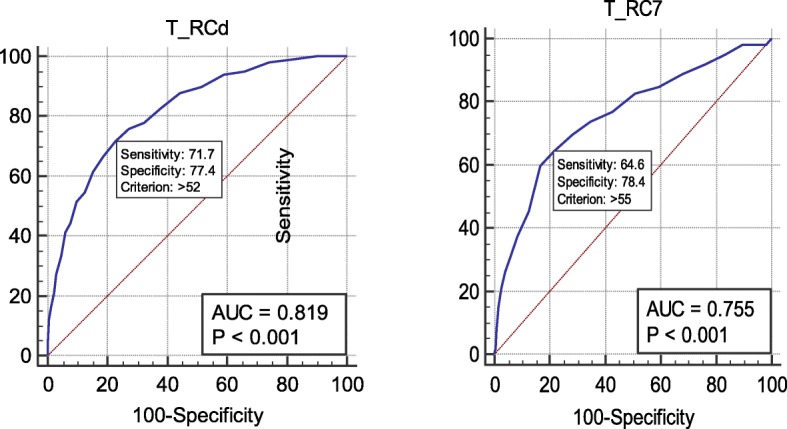
ROC curve of the Demoralization (RCd) in suicidal high suicidality group. AUC = 0.819 (95% CI 0.811 to 0.828), ROC: Receiver Operating Characteristics, AUC: Area Under the Curve

Among the RC scales, Demoralization (RCd) and Dysfunctional Negative Emotions (RC7) showed the largest significant difference between the control group and three suicide risk groups, and we conducted ROC analysis to determine how accurately the two scales discriminate the groups with regard to suicide risk. For the Demoralization (RCd), the area under the curve (AUC) of the ROC, which indicates diagnostic accuracy, was highest at 81.9% (*p* < 0.001) for the high-risk group, indicating a moderate accuracy level, followed by 78.9% for the moderate-risk group and 71.1% for the low-risk group. For Negative Emotions (RC7), the AUC was 75.5% for the high-risk group, 74.1% for the moderate-risk group, and 67.3% for the low-risk group, suggesting moderate predictive power of Negative Emotions (RC7) as well (Table 4). For the Demoralization (RCd) of the high-risk group with the highest predictive accuracy, the cutoff score was 52, and sensitivity and specificity were 71.7 and 77.4%, respectively (Fig. 2). AUC scores were lower for Negative Emotions (RC7) than for Demoralization (RCd), indicating fair accuracy; however, the Negative Emotions (RC7) cutoff score for the high suicide risk group was 55, and the sensitivity and specificity of the scale were 64.7 and 78.4%, respectively.

**Table 4:** The sensitivity and specificity for different cutoff points of between suicidal risk group and RCd, RC7

	Cut off	AUC	Sensitivity	Specificity	*P* value
Suicidal Low risk					
RCd	45	0.711(0.701–0.721)	73.06(69.2–76.7)	57.35(56.2–58.5)	< 0.0001
RC7	49	0.673(0.663–0.684)	67.81(63.7–71.7)	58.70(57.6–59.8)	< 0.0001
Suicidal Moderate risk					
RCd	49	0.789(0.780–0.798)	74.68(69.5–79.4)	68.86(67.8–69.9)	< 0.0001
RC7	51	0.741(0.731–0.751)	70.83(65.4–75.8)	66.07(65.0–67.1)	< 0.0001
Suicidal High risk					
RCd	52	0.819(0.811–0.828)	71.72(61.8–80.3)	77.36(76.4–78.3)	< 0.0001
RC7	55	0.755(0.746–0.765)	64.65(54.4–74.0)	78.38(77.4–79.3)	< 0.0001

## Discussion

This study investigated the utility of the MMPI-2-RF as a suicide risk assessment tool and the variation in the RC Scales according to suicide risk.

The RC Scales varied in score elevation according to suicide risk level. For all three suicide risk groups, the RC Scale scores were significantly higher than in the control group. This suggests that various psychopathological characteristics are involved in suicide, and is consistent with previous findings [13]. In particular, the Demoralization (RCd) and Dysfunctional Negative Emotions (RC7) Scale scores varied according to suicide risk level as well as between controls and those at risk of suicide. In all three suicide risk groups, the Demoralization (RCd) scores were highest, followed by the Dysfunctional Negative Emotions (RC7) scores. For both Demoralization (RCd) and Dysfunctional Negative Emotions (RC7), the difference between the three groups was significant, but the difference between the moderate- and the high-risk groups was not. This result can be interpreted as reflecting the characteristics of the MINI which was used for suicide risk assessment. In the MINI, those who planned, attempted, or considered suicide within a month are classified into the moderate- and the high-risk groups.

The Demoralization (RCd) and Dysfunctional Negative Emotions (RC7) may only be able to discriminate the low-risk group from higher risk groups. Regarding the Demoralization (RCd), high scale scores indicate demoralization, which is manifested by dissatisfaction and unhappiness in life as a whole, a sense of helplessness and inefficiency, and a pessimistic attitude toward the future. Elevation in Demoralization (RCd) is often accompanied by “patienthood,” including hopelessness, pessimism, self-degradation, depression, suicidal ideation, and somatic complaints [12]. The Dysfunctional Negative Emotions (RC7) indicates dysfunctional Negative Emotions, including anxiety, anger, and fear. Those with high Dysfunctional Negative Emotions (RC7) scores are likely to develop an anxiety disorder, and experience excessive rumination and a sense of guilt [10]. The Demoralization (RCd) and Negative Emotions (RC7) scores indicate issues originating from emotional problems; therefore, emotional pain can be considered a major factor that distinguishes between normal and suicidal groups, The psychiatric theory of Shneidman (1993) and the interpersonal-psychological theory by Joiner (2005) state that suicidal behavior results from psychological pain caused by frustrated need [41, 42]. The escape theory of Baumeister (1990) maintains that psychological pain is derived from negative effects generated by aversive self-awareness [43]. Each theory suggests a different cause for psychological pain but the theories agree that the desire of suicide stems from psychological pain [44]. Moreover, the “ideation-to-action” theory of Klonsky and May (2014) expresses that suicidal ideations grow and progress to strong ideations when the amount of pain is greater than that of connectedness to others, and the ideation proceeds to an attempt depending on the possibility of a suicide attempt [45]. Therefore, the group with a high risk of suicide would have a high level of a combination of pain and hopelessness. Therefore, this emotional pain can be presented by RCd and RC7.

Moreover, the odds ratios for the two scales increased with the suicide risk indicated by the group membership, suggesting that the effect of mental pain on suicide increases as the degree of mental pain increases. The results also indicate the scales’ discriminatory power in distinguishing between those at risk and controls.

The high-risk suicide group scored higher than the low-risk group on all RC Scales except RC9, and was influenced by more RC Scales compared to the low/moderate risk groups, suggesting the presence of more psychopathological personality factors. The findings suggest that a higher suicide risk level is associated with the presence of various psychopathic personality factors that increase suicide risk [11]. However, the difference between the moderate- and the high-risk groups was large for the Demoralization (RC1), Low Positive Emotions (RC2), Cynicism (RC3), Antisocial Behavior (RC4), and Ideas of Persecution (RC6) scales, and Somatic Complaint (RC1), Low Positive Emotions (RC2), Antisocial Behavior (RC4), and Ideas of Persecution (RC6) were significant in the regression analysis, suggesting that the Demoralization (RCd) and the four RC scales reflect significant psychopathological issues for the high-risk suicide group. The Somatic Complaint (RC1), Low Positive Emotions (RC2), Antisocial Behavior (RC4), and Ideas of Persecution (RC6) Scales suggest that suicide planning and suicide attempt in the high-risk suicide group is a combined effect of emotional, behavioral, and cognitive characteristics.

The moderate-risk group differed significantly from the low/high-risk groups on the RCd and RC1. The low-risk group showed an overall elevation in the RC Scales compared to the control group and was significantly influenced by the Demoralization (RCd), Antisocial Behavior (RC4), and Aberrant Experiences (RC8) scores. This suggests that even the low-risk group may have distinct behaviors and perceptual experiences such as antisocial behaviors (RC4). Along with the unusual thinking and perceptual experience of Aberrant Experiences (RC8), the common traits of the both scales (easily changing emotions) can be considered a characteristic that predicts suicide.

The significantly high Demoralization (RCd) and Negative Emotions (RC7) scores and ORs demonstrate that they are important psychopathological predictors of suicide. Although the Demoralization (RCd) and Negative Emotions (RC7) scores are highly correlated, their content and experiential correlates are distinct; therefore, distinct emotional and experiential factors are likely involved in the etiology of suicide [9].

According to the results of the ROC curve analysis designed to determine the diagnostic accuracy of the Demoralization (RCd) and Negative Emotions (RC7) scales for suicide risk, the AUCs for both Demoralization (RCd) and Negative Emotions (RC7) varied according to suicide risk. The AUCs for the Demoralization (RCd) were highest for the high-risk group, followed by the moderate-risk group (0.789) and the low-risk group (0.711). Similarly, the AUCs for the Negative Emotions (RC7) were highest for the high-risk group (0.755), followed by the moderate-risk group (0.741) and the low-risk group (0.673). The results suggest an overall moderate accuracy level except for Negative Emotions (RC7) in the low-risk group [16]. The Demoralization (RCd) had the largest AUC, classifying 77% of the high-risk group. In this study, the scale that was most effective in discriminating between the high- and the moderate-risk for suicide groups was the Demoralization (RCd) followed by Negative Emotions (RC7) [17].

The findings of this study have significant implications for suicide risk assessment, as we segmented the suicide-risk group in a sample of college students in a non-clinical setting, and analyzed the psychopathological factors that affect suicide risk using the widely used MMPI-2-RF RC Scales in suicide risk assessment.

This study has some limitations. First, study participants were a non-clinical sample of college students. Patients with mental illness are 3 to 12 times more likely to commit suicide when compared with other patients. Individuals with depression are more likely to experience suicidal death than the general population, and among psychiatric patients, those with suicidal ideation along with substance abuse and impulse control disorder have a high risk of attempting suicide. Accordingly, the clinical population is expected to manifest more diverse and complex psychopathological personality characteristics when compared with the non-clinical population, suggesting the need for further research involving a clinical population. Further research is needed with clinical populations.

Second, all study data were obtained from self-report tests, limiting the reliability of responses.

The suicide risks and pathological characteristics of individuals should be assessed using various tools and methods, such as direct and indirect measures designed to determine suicide tendencies, assessment methods used by clinicians, and psychological tests, to identify individual psychopathological personality characteristics.

Third, detailed data on suicide are needed. The purpose of this study was to evaluate the risk of suicide using a module of the MINI, but no detailed information about the criticality of suicide risk, method or frequency was obtained. Therefore, an assessment tool for the accurate segmentation of suicide risk groups based on a greater specificity of suicidal ideation, attempts, and planning is required.

Notwithstanding these limitations, this study has particular significance for segmenting and analyzing suicide risk using the MMPI-2-RF RC Scales. In this study, psychopathological factors affecting suicide were also identified by examining the effects of the RC Scales in discriminating between different levels of suicide risk. In most studies on suicide risk and tendency using the MMPI-2-RF, the authors recognized a correlation with suicide through use of the RC scale (e.g., RCd and RC9) based on an emotional theory or an interaction between the scales. In contrast, we identified the RC scales presenting differential effects on each suicide risk group; only the RC9 had no effect on the high-risk group. Our findings disagree with precedent studies stating that RC9 is the major scale which influences suicide risk. Our research implies that stratification of the risk groups allows for more specific assessments. Furthermore, we found the reference point of RCd and RC7 (the most influential scale for all suicide risk groups) in the non-clinical population by ROC analysis.

## Conclusions

The study examined the differences between the RC Scales according to suicide risk level. Compared to a healthy control group, all three suicide risk groups’ scores were higher on the MMPI-2-RF RC Scales in general, suggesting that various psychopathological characteristics are involved in suicide risk. It was also found that more psychopathological factors, and therefore more risk factors, influenced suicide-related issues in the groups with a higher risk for suicide. Even the low-risk for suicide group differed from the healthy control group for emotional factors and antisocial behaviors. The high-risk suicide group was differed to a large extent from the other groups on the Demoralization (RCd) and Negative Emotions (RC7) scales, suggesting their utility in screening for suicide risk.

## Data Availability

The datasets analyzed during the current study are not publicly available due Personal Information Protection Act of Korea.

## Abbreviations

ANOVA: Analysis of variance
MMPI-2-RF: Minnesota Multiphasic Personality Inventory-2 Restructured Form
OR: Odds ratios
PHQ-9: Patient health questionnaire-9
RC: Restructured Clinical Scales
